# Reciprocal impact of mental health and quality of life in children and adolescents—a cross-lagged panel analysis

**DOI:** 10.3389/fpsyg.2025.1444524

**Published:** 2025-03-26

**Authors:** Markus A. Wirtz, Janine Devine, Michael Erhart, Franziska Reiß, Maren Böcker, Anja A. Schulz, Fionna Zöllner, Ann-Kathrin Napp, Ulrike Ravens-Sieberer, Anne Kaman

**Affiliations:** ^1^Research Methods in Health Science, University of Education, Freiburg, Germany; ^2^Department of Child and Adolescent Psychiatry, Psychotherapy, and Psychosomatics, University Medical Center Hamburg-Eppendorf, Hamburg, Germany; ^3^Alice Salomon University of Applied Science, Berlin, Germany; ^4^Child Neuropsychology Section, Department of Child and Adolescent Psychiatry, University Hospital Aachen, Aachen, Germany; ^5^Institute of Medical Psychology and Medical Sociology, University Hospital of RWTH Aachen University, Aachen, Germany

**Keywords:** quality of life, mental health, children’s health, adolescent’s health, COVID-19 pandemic

## Abstract

**Introduction:**

A thorough understanding of the interplay of mental health (MH) and quality of life (QoL) is essential to describe, understand and support the healthy development of children and adolescents. The aim of the study is to analyze the reciprocal and predictive relationship between psychosomatic symptoms, MH problems and QoL in children and adolescents during the COVID-19 pandemic using a cross-lagged panel analysis.

**Methods:**

Data of *n* = 323 children and *n* = 421 adolescents were collected at five measurement points from spring 2020 to autumn 2022 within the population-based longitudinal German COPSY study. Parent proxy ratings were assessed using the KIDSCREEN-10 index (QoL), the Strengths and Difficulties Questionnaire (SDQ; internal and external MH symptoms) and the Health Behavior in School-aged Children Symptom Checklist (HBSC-SCL; psychosomatic symptoms). Adolescents also self-rated the KIDSCREEN-10 Index and the HBSC-SCL. Cross-lagged-panel models, which offer higher internal validity than traditional cross-sectional and longitudinal analyses, were estimated using structural equation modeling (maximum likelihood).

**Results:**

Different prediction models proved to be valid for children vs. adolescents (Δχ^2^_*df* = 48_ = 167.84, *p* < 0.001). For children, QoL did not cross-predict MH indicators (Δχ^2^_*df* = 12_ = 15.53, *p* > 0.05), but was the time-lagged criterion variable most strongly predicted by them (Δχ^2^_*df* = 12_ = 71.58, *p* <0.001). For adolescents, self-reported QoL cross-predicted psychosomatic symptoms (Δχ^2^_*df* = 3_ = 14.22, *p* < 0.001). For both children and adolescents, internalizing MH problems cross-predicted QoL and psychosomatic symptoms (Δχ^2^_*df* = 3_ = 9.58–13.69, *p* < 0.001).

**Discussion:**

Psychosomatic and psychological MH symptoms were proven to be significant time-lagged predictors of QoL, particularly in children. Thus, they can serve as preceding indicators for the development of QoL. Since the cross-lagged panel approach provides a higher internal validity than e.g., cross-sectional data analyses, our findings may contribute to an enhanced understanding of mental development processes and, thus may provide evidence for targeted support of healthy development under demanding conditions such as the COVID-19 pandemic.

## 1 Introduction

The COVID-19 pandemic fundamentally changed the lives and opportunities for experience and behavior of children and adolescents. The exceptional importance of protection against the risk of infection risks in everyday life, which was accompanied by restrictions of school attendance as well as leisure and further social activities, affected mental health (MH; Kauhanen et al., 2023; Orban et al., 2024; Wolf and Schmitz, 2023). To determine the impact of the straining conditions on the health of children and adolescents in the pandemic, changes in Quality of Life (QoL) and MH indicators have been investigated intensively. Most studies have shown a systematic decline in QoL (Belanger et al., 2021; Chen et al., 2021; Genta et al., 2021; Ma et al., 2021; Mastorci et al., 2021; Ravens-Sieberer et al., 2023a, 2023b; Thorisdottir et al., 2023). For MH problems, an increase in internalizing (8 of 21 studies) or externalizing symptoms (8 of 13 studies) was found (Wolf and Schmitz, 2023). Contradictory findings were obtained for psychopathology and psychosomatic symptoms (Schlack et al., 2023), as both increasing (Chen et al., 2021; Larsen et al., 2022; Ravens-Sieberer et al., 2023a, 2023b; Theuring et al., 2022; Thorisdottir et al., 2023) and decreasing (Van der Laan et al., 2021) symptoms have been reported. These developments have been demonstrated primarily in the early period of the pandemic (the first 1.5 years) within cross-sectional studies. A systematic review of longitudinal studies showed that children and adolescents experienced increased MH problems, particularly internalizing symptoms such as anxiety and depression (Orban et al., 2024). Most analyses focus on general adaptation to the pandemic, while few studies use longitudinal mixed-distribution models to examine subgroup differences in MH and QoL (Kaman et al., 2024; Wang et al., 2022). While homogeneous development can be assumed for QOL, heterogeneous development patterns were found for externalizing and internalizing symptoms as well as for psychosomatic symptoms, which can be interpreted in terms of increased resilience or vulnerability to stress and symptom progression (Kaman et al., 2024). Overall, the study findings do not clearly determine whether, and to what extent, changes in the MH of children and adolescents were influenced by (i) the direct health risks posed by the COVID-19 virus or (ii) the restrictions imposed on individual and social life. Additionally, the degree to which each factor contributed to these changes remains uncertain.

### 1.1 Association of MH and QoL in children and adolescents

To better understand the health development of children and adolescents and to provide them with more targeted interventional support under demanding living conditions, empirical evidence on whether, how and to what extent MH contributes to the individual development of QoL—or vice versa—would be valuable (Belanger et al., 2021; Chen et al., 2021; Groß et al., 2024; Orban et al., 2024; Ravens-Sieberer et al., 2023a, 2023b; Thorisdottir et al., 2023; Wolf and Schmitz, 2023). In the context of the extensive research on the average changes in MH and QoL of children and adolescents over the course of development, such potential reciprocal dependencies between MH and QoL are always subject of assumed theoretical models and discussions (Connell et al., 2014). Based on socio-cognitive behavioral models, Mierau et al. (2020) argue that mental disorders significantly affect the QoL of those affected in the long term. MH problems are associated with maladaptive thinking patterns (e.g., rumination), negative self-talk, emotions, and avoidance behaviors that may impair their development and experiences, which are associated with QoL (Bertha and Balazs, 2013; Tsomokos and Flouri, 2024). Moreover, adolescents facing chronic MH challenges are less resilient to environmental stressors, such as peer conflicts or academic pressures, which may negatively affect their QoL development (Mc Elroy and Hevey, 2014). According to the self-determination theory, limitations of autonomy, experience of competence, and social connectedness are unfavorable motivational prerequisites for self-efficacy and positive life experiences (Stanton et al., 2020). QoL is generally considered to be influenced by prior physical health and MH aspects (Bullinger and Quitmann, 2014). According to the definition of the WHO, health-related QoL is understood as a multidimensional construct comprising subjectively assessed aspects of physical, psychological, mental and social well-being. The KIDSCREEN instrument defines physical and psychological well-being (incl. mood and emotions, self-perception), autonomy and relationship with parents, relationship with peers, social support and the school environment as structural components (Ravens-Sieberer and The European KIDSCREEN Group, 2006).

Resilience refers to the ability to adapt, recover, and thrive amid adversity. It is a dynamic process of maintaining or restoring MH under psychosocial risks (Ronen, 2021). Resilient children and adolescents adapt effectively to challenges with solution-oriented coping skills (Groß et al., 2024), including emotional and cognitive skills like self-efficacy, problem-solving, and optimism, as well as the ability to utilize social resources (Mesman et al., 2021). For example, in observational studies, resilience of children and adolescents is assumed as a key protective factor for the development of their MH and QoL (Barbieri et al., 2023; Celebre et al., 2021; Connell et al., 2014; Kauhanen et al., 2023; Mesman et al., 2021; Montero-Marin et al., 2023; Olsavsky et al., 2024; Tal-Saban and Zaguri-Vittenberg, 2022; Wolf and Schmitz, 2023). But resilience itself is also considered to be significantly characterized or influenced by MH resources and social resources in the environment (Barbieri et al., 2023; Mesman et al., 2021; Montero-Marin et al., 2023; Olsavsky et al., 2024; Tal-Saban and Zaguri-Vittenberg, 2022). MH is thus regarded as an important resource enabling children and adolescents to mitigate and cope with stress and to develop and activate functional coping strategies (Groß et al., 2024). Hence, it is assumed that MH serves as decisive preventive factor in determining whether they can act resiliently to maintain or improve their future QoL development (Mesman et al., 2021; Mikkelsen et al., 2022; Montero-Marin et al., 2023; Olsavsky et al., 2024; Orban et al., 2024; Ravens-Sieberer et al., 2022a, 2022b, 2023a, 2023b; Tal-Saban and Zaguri-Vittenberg, 2022). Additionally, living conditions of children and adolescents vary based on family and social differences (Barbieri et al., 2023; Connell et al., 2014; Mesman et al., 2021; Tal-Saban and Zaguri-Vittenberg, 2022). Thus, mental resources are crucial for children and adolescents to react to stress constructively, so that their QoL is not adversely affected. Conversely, QoL impairments may also impact health-related lifestyle and associated MH aspects (Montero-Marin et al., 2023; Olsavsky et al., 2024; Ravens-Sieberer and The European KIDSCREEN Group, 2006; Schlack et al., 2023).

Children’s and adolescents’ everyday lives and MH were affected differently by the pandemic. For children, family circumstances were crucial, as parents played a more active role, reducing children’s independence in daily routines and social life (Krijger et al., 2022; Soejima, 2021). School closures and limited social interaction led to increased isolation, anxiety, and insecurity, impacting the social and emotional development (Twum-Antwi et al., 2020). Parental stress also influenced family dynamics (Krijger et al., 2022). Adolescents faced distinct challenges tied to identity formation and social bonding (Annam et al., 2022; Creswell et al., 2021; Fong and Iarocci, 2020). Literature highlights loneliness, depression, and future-related fears (Gniewosz, 2023; Jost et al., 2023; Kayaoğlu and Başcıllar, 2022). Self-directed and exploratory forms of behavior, which are central to identity formation in adolescence, were particularly affected. Adolescents were also more exposed to media influences that exacerbated additional stress and uncertainty about the future during the pandemic (Marciano et al., 2022).

This study examines the link between internalizing and externalizing symptoms, behaviors, and psychosomatic symptoms with children’s and adolescents’ QoL during the COVID-19 pandemic. Internalizing behaviors, assessed via the Strength and Difficulties Questionnaire (SDQ; Haugland and Wold, 2021; Klasen et al., 2003), involve emotional problems like anxiety, sadness, and stress-related symptoms such as headaches, stomach aches, as well as social difficulties. They often manifest as worry, low mood, and isolation. Externalizing behaviors include aggression, defiance, rule-breaking (e.g., lying, fighting, stealing), hyperactivity, impulsivity, and inattention. These are more visible in social settings. Psychosomatic symptoms arise from psychological stress, anxiety, or emotional strain, leading to headaches, nausea, fatigue, muscle pain, dizziness, and sleep disorders (Gariepy et al., 2016; Goodman, 1997). Such symptoms often reflect an inability to express emotional distress. Since interventional studies on effects in natural settings are not feasible for children’s and adolescents’ long-term health development, the according arguments are based primarily on cross-sectional and longitudinal observational study findings. Thus, empirical sound evidence on directed temporal dependencies and directions of influence between MH indicators and QoL is limited (Kenny, 2004; Singer and Willett, 2023).

### 1.2 Adopting cross-lagged-panel-designs to analyze the reciprocal time-shifted prediction between MH aspects and QoL

**Figure 1:**
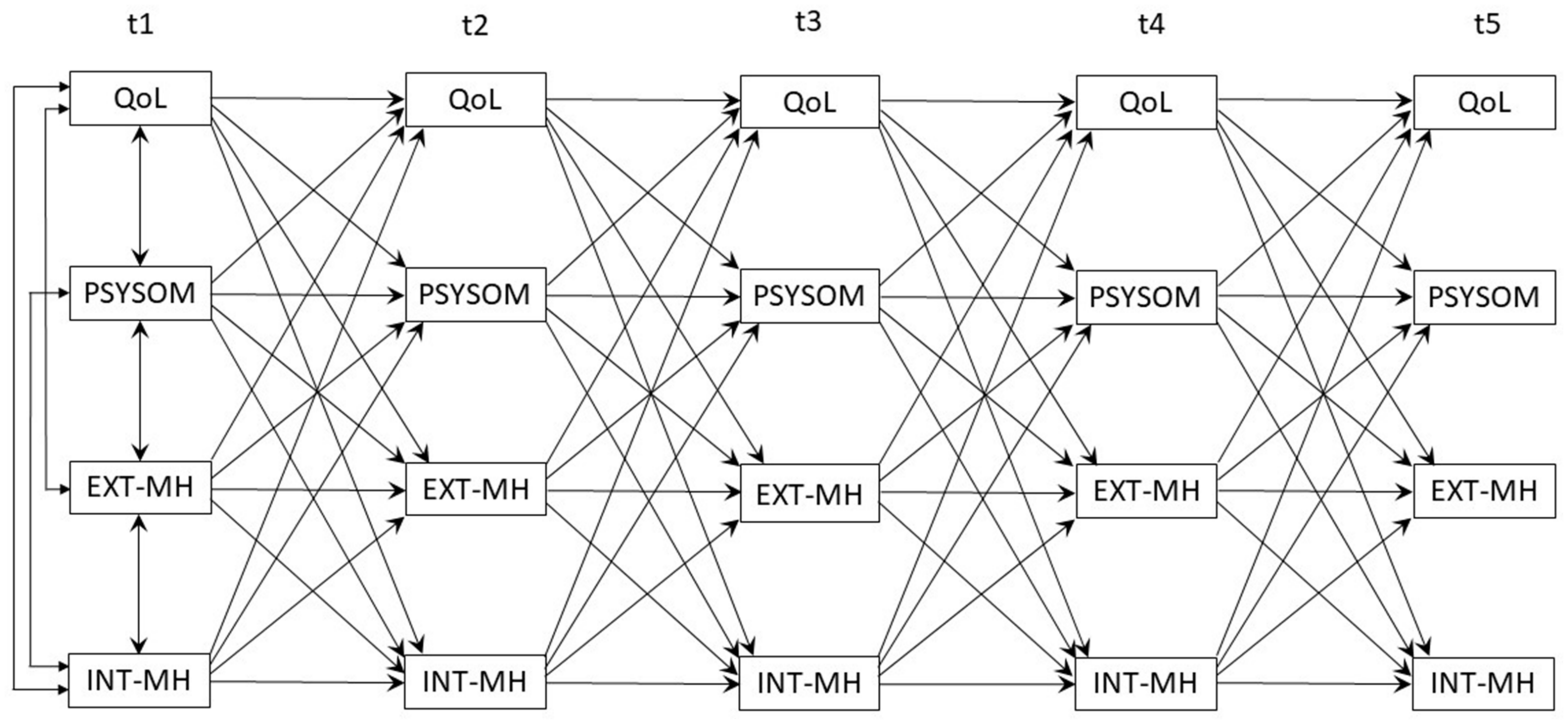
Original definition of the cross-lagged-panel model.

In this study, the cross-lagged panel design (CLPD) methodology will be applied for the first time to the best of our knowledge to analyze directional dependencies between MH aspects and QoL during the course of the pandemic. CLPDs are considered superior to purely cross-sectional or longitudinal analyses in terms of their enhanced internal validity, as (i) cross-sectional and time-shifted dependencies between characteristics and (ii) longitudinal courses of the individual characteristics are modeled in an integrated manner (see Figure 1) (Finkel, 1995; Kendel et al., 2010; Kenny, 2004; Lüdtke and Robitzsch, 2022; Orth et al., 2021; Robitzsch and Lüdtke, 2024; Selig and Little, 2012; Singer and Willett, 2023; Zyphur et al., 2019). Accordingly, CLPDs are suitable to determine the predictive power of each variable at time ti for the same variable (i.e., stability of the according trait) as well as for the other variables (i.e., time lagged cross-prediction regarding other traits) at t_i + 1_. This combined analysis allows for determining which traits are time-shifted predictive for the expression of other traits in the future. Significant time-shifted cross-prediction provides information on whether a trait can be used as a temporally preceding predictor (in the sense of an early warning indicator). In addition to such a diagnostic implication, corresponding time-shifted cross-predictive effects may also be interpreted as indications of causal directions: a temporally preceding cross-predictor variable (at time t_i-1_) can be regarded as an antecedent condition for the temporally following cross-criterion (at time ti). However, it must be noted that any causal impact interpretation would be based on the assumption that all characteristics relevant to the effect structure would have been included in the structural model. Otherwise, potential confounding effects may limit the evidence for causal linkage (Finkel, 1995; Kenny, 2004; Robitzsch and Lüdtke, 2024; Selig and Little, 2012; Singer and Willett, 2023; Zyphur et al., 2019). Given potential differences in MH and QoL development (Farrell et al., 2023; Montero-Marin et al., 2023; Olsavsky et al., 2024; Otto et al., 2017), this study explores moderating effects between children and adolescents.

Since the primary research objective is to understand stable, inter-individual differences in longitudinal associations rather than dynamic processes within individuals, the applied cross-lagged panel design (CLPD) focuses on the effects between individuals (Robitzsch and Lüdtke, 2024). Thus, the relative expression of individual characteristics compared to the other participants is considered crucial for predicting future development. This approach is particularly appropriate in contexts where the theoretical emphasis is on traits, enduring dispositions, or contextual factors that vary systematically across individuals and persist over time. Applying a mixed-distribution modeling approach (i.e., random-intercept cross-lagged panel model; RI-CLPM; Hamaker et al., 2015; Hamaker, 2023; Lucas, 2023) would additionally allow to account for intraindividual variability and temporal dynamics. This would provide additional insight into the intraindividual variability of the state variables at a specific point in time and enable changes in the individuals’ own state to be predicted. However, since these effects are not the subject of the research hypotheses, which focus in particular on moderation effects between children and adolescents a between effects approach is chosen.

### 1.3 Present study and research aims

The aim of this study is to obtain evidence of mutual time-shifted cross predictions between QoL, internalizing and externalizing MH problems as well as psychosomatic symptoms in children and adolescents. To this ends, this study uses longitudinal data collected at five measurement time points over a period of three pandemic years from the German population-based COVID-19 and Psychological Health (COPSY) study (Ravens-Sieberer et al., 2022a,b, 2023a,b). The following study hypotheses will be examined:

*Hypothesis 1*: *Fit of the CLPD-model*: The CLPD model fits the assumed cross-prediction data structure of psychosomatic symptoms, externalizing and internalizing MH problems, and QoL appropriately.

*Hypothesis 2*: *MH cross predicts future QoL*: Psychosomatic symptoms as well as externalizing and internalizing MH problems determine QoL at the following measurement time in terms of cross-prediction.

*Hypothesis 3*: *Cross predictions differ for children and adolescents*: For children and adolescents, different time-lagged relationships apply to psychosomatic symptoms, externalizing and internalizing MH problems, and QoL.

## 2 Methods

### 2.1 Study design and sample characteristics

**Figure 2:**
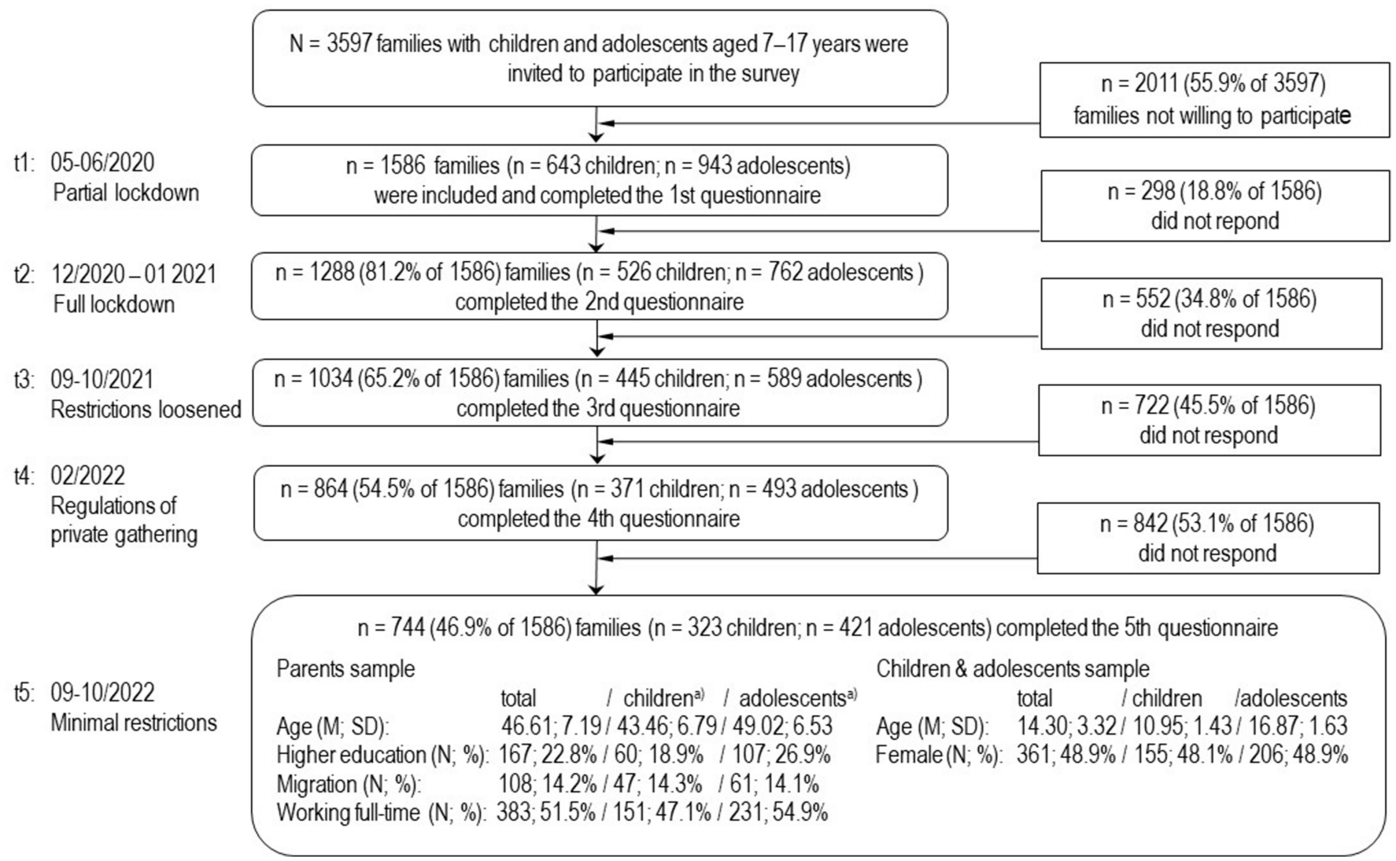
Flow chart of the families participating in the COPSY study.

The population-based longitudinal German COPSY study (Ravens-Sieberer et al., 2022a, 2022b) assessed children, adolescents, and their parents/caregivers at five assessment points: At the start of the COVID-19 pandemic, when there was a partial lockdown (t_1_: 05–06/2020); during the first pandemic winter with a full nationwide lockdown (t_2_: 12/2020–01/2021); after the summer in the second year, when infection rates were low, and restrictions loosened (t_3_: 09–10/2021); at the end of the second pandemic winter, when there were still regulations of private gatherings (t_4_: 02/2022); and in autumn of year three of the pandemic, when only minimal restrictions remained (t_5_: 09–10/2022). The intervals between the survey periods varied between 6 and 8 months due to the organization of the data collection. Families were recruited through a population-based approach from an online panel using quota sampling to approximate the socio-demographic characteristics of the German population in the study sample. Parents were initially approached online via e-mail, informed about the study objectives, and asked to provide their informed consent. After completing the online survey themselves, parents were instructed to forward the survey link to their child. Adolescents aged 11 years and older completed self-reported questionnaires, while data for younger children (7–10 years) were collected via parent proxy-report only. Families who participating in the first wave of data collection were re-invited to each follow-up assessment. Figure 2 depicts the participants flow over the course of the study. Of the *N* = 3597 families initially contacted, *n* = 1,586 families (44.1% of 3,597) answered the questionnaire at t_1_. Of those, *n* = 744 families (53.1% of 1,586; *n* = 323 children aged 7–10 years, *n* = 421 adolescents aged 11–17 years) responded at all five assessment points. *n* = 842 were not included in the analysis as they dropped-out earlier (at t_2_ *n* = 298, at t_3_ *n* = 255, at t_4_ *n* = 170, at t_5_ *n* = 120).

The *n* = 744 children and adolescents who took part in all five measurement points were slightly older (1.3 years) than those not included. Furthermore, parental education level was slightly lower, and participants were less likely to have a migration background (small effect sizes). No significant differences prevailed for gender, single vs. co-parenting and parental employment. Significantly—but also with only small effect sizes—lower values apply for externalizing psychological problems (at three of five measurement points) and for internalizing psychological problems and psychosomatic complaints (at one of the five measurement points) in the final analysis sample.

Although correlations with sociodemographic variables and MH indicators proved to be rather weak, they suggest that the drop-out occurred not completely at random (MCAR; Graham, 2009; Wirtz, 2004). Although likelihood-based imputation methods (e.g. FIML) can provide unbiased estimates for MAR processes, they require sufficient data on dropout-related characteristics, which were not comprehensively recorded in this study (Graham, 2003; Enders, 2008). In the COPSY study, data were collected under pandemic conditions, with survey waves differing by seasons and social contact rules (Enders, 2008). Since covariates were only collected once, no estimation-based approach was used. Weak correlations with analysis variables further indicated that nonresponse processes could not be appropriately retraced (Graham, 2009). Instead of using imputation models with limited validity, only data of families that consistently participated were analyzed (Kaman et al., 2024). Thus, it should be kept in mind that the results only apply to families that participated throughout the pandemic period.

### 2.2 Measures

The study used internationally established and validated assessment instruments. QoL was measured via the KIDSCREEN-10 Index (internal consistency, Cronbachs *α*: 0.82 (self-ratings); 0.78 (proxy-ratings); retest reliability, ICC: 0.70 (self-ratings); 0.68 (proxy-ratings); Ravens-Sieberer and The European KIDSCREEN Group, 2006). Frequencies of psychosomatic symptoms were measured via the Health Behavior in School-Aged Children Symptom Checklist (HBSC-SCL; internal consistency, Cronbachs *α*: 0.73; retest-reliability (4–6 months): *r_tt_* = 0.62; Goodman, 1997, 2001). MH problems were assessed using the Strengths and Difficulties Questionnaire (SDQ; Haugland and Wold, 2021; Klasen et al., 2003; internal consistencies of total score, Cronbachs *α*: 0.76–0.79; retest: *r_tt_* = 0.60–0.73; Lohbeck et al., 2015), which provides externalizing (hyperactivity and conduct problems) and internalizing (emotional and peer problems) scores. Parent-reported questionnaire data were available for n = 744 children and adolescents for all assessment points. Additionally, self-reported data from the KIDSCREEN-10-Index and HBSC-SCL were obtained for *n* = 421 adolescents. A more detailed description of the study design and administered instruments are reported elsewhere (Ravens-Sieberer et al., 2022a, 2022b).

### 2.3 Statistical analysis

The time-lagged dependencies of QoL (KIDSCREEN-10 Index), psychosomatic symptoms (HBSC-SCL), externalizing and internalizing MH problems (SDQ) were analyzed in a CLPD (Finkel, 1995; Kenny, 2004; Selig and Little, 2012; Singer and Willett, 2023) using structural equation modeling (SEM; Kline, 2023). In contrast to classical correlation and regression analysis methods, SEM offers the possibilities (a) to model multivariate dependencies of several correlated criterion variables simultaneously, and (b) to use dependent criteria as predictors for subsequent criteria. In addition, SEM allows testing the data fit of the assumed multivariate model structure. At t_1_, the variables were defined as correlated exogenous variables, because medium to high associations between MH aspects and QoL are consistently documented in the literature (Celebre et al., 2021; Kaman et al., 2024; Kauhanen et al., 2023). For the subsequent assessment times t_2_...t_5_, the prior values of the variables themselves (stability of each construct) and of the other three constructs (cross predictions) were modeled as predictors (Figure 1).

Model parameters were estimated using the maximum likelihood algorithm (ML) implemented in AMOS 29.0. For valid estimation of the SEM using the maximum likelihood approach, the assumption of multivariate normally distributed data should be fulfilled. Since the Kolmogorov-Smirnoff test already indicates violations for marginal deviations in large samples (*N* > 300) due to high test power, recommendations of Kline (2023) are applied: Estimates can be considered robust if skewness is below 3 and kurtosis below 8. Kolmogorov–Smirnov test showed a deviation from the normal distribution assumption for all four analysis characteristics QoL, psychosomatic symptoms, external and internal MH for all five measurement time points (*p* < 0.001). However, values of skewness 0.433–1.332 and kurtosis 0.031–2.477 clearly undercut Kline’s critical thresholds, indicating that the assumptions can be regarded as not critically violated.

Although a significant *χ^2^*-value indicates deficiencies in the model fit, its validity is limited due to its overly sensitivity to marginal misspecifications, especially for *n* > 300. Accordingly, the incremental fit measures Tucker-Lewis Index (*TLI*) and Comparative Fit Index (*CFI*) as well as Root Mean Square Error of Approximation (*RMSEA*) were used for assessing model fit (acceptable/good fit: *TLI*, *CFI* > 0.95/ > 0.97; *RMSEA* < 0.08 / < 0.05) (Hu and Bentler, 1999; Kline, 2023; Schermelleh-Engel et al., 2003).

Study hypotheses were tested by means of hierarchical, nested model comparisons (Kline, 2023). For nested models and when using ML-estimation the difference Δχ^2^ between the *χ^2^* values of the unrestricted model (free parameter estimation) and the restricted model (tested parameters restricted to 0) is itself *χ^2^*-distributed (*df* = difference of the df of both models). A significant Δχ^2^-value indicates that the restrictions are associated with a systematic loss of information and are therefore inappropriate (Kline, 2023). To test the directed time-shifted cross prediction of two analysis characteristics (e.g., psychosomatic symptoms → QoL), the corresponding four model paths t_1_ → t_2_, t_2_ → t_3_, t_3_ → t_4_ and t_4_ → t_5_ were tested simultaneously by restricting all four parameters to 0 in the nested model specification. This overall interval testing was considered adequate, because there were no assumptions about the difference in cross-predictions between the intervals for the same combination of variables. Moreover, testing these overall hypotheses considerably increased test economy and test power (Ellis, 2010). To examine general moderating effects of age-groups children vs. adolescents, each of the 48 cross-prediction paths were set equal between children and adolescents in an overall model (group-invariant estimation of the cross-predictions).

Since the validity of the model estimates could be impaired by varying construct meanings between measurement points in time, the invariance of the measurement models between the five measurement points in time should be checked (Putnick and Bornstein, 2016). To this end, a CFA is defined for each of the measurement scales KIDSCREEN (Qol), HBSC-SCL (psychosomatic symptoms) and internal and external MH according to SDQ. Within the model specifications each construct is specifically defined for each measurement time point (e.g., Qol_t1...Qol_t5) by assigning only the time point-specific item measurements to these (Speyer et al., 2023). In the CFA model, both the five instrument-specific constructs defined in this way and the item-specific measurement error terms are assumed to be correlated over t_1_ to t_5_. These models are estimated with item-specific unstandardized loadings, once free and thus measurement time-specific (reference model) and once as equal for each item at all five times point, i.e., restricted as invariant over time (tau-equivalent estimation). As these are nested models, significant Δχ^2^-tests indicate a violation of time invariance (Putnick and Bornstein, 2016). However, as this test indicates deviations even for marginal violations in samples with N > 300, the *RMSEA* offers a more valid comparative measure, as it adjusts the χ*^2^*-value to correct not only for the sample size but also for the model complexity. The information-theoretical measure Bayesian information criterion (*BIC*; lower values indicate better model quality) and the parsimony-adjusted *PCFI* (higher values indicate better model quality) allow a meaningful comparison of models of different complexity. The difference in the normalized fit index can be used as a measure of the absolute contrast of the model fit: The higher the *ΔNFI* of tested models, the greater the difference in data compatibility between the comparison models (Kline, 2023; Schermelleh-Engel et al., 2003).

## 3 Results

*Hypothesis 1—Fit of the CLPD-model*: The initially defined CLPD model of parent-reported data proved to be insufficiently consistent with the data in the whole study sample (*χ^2^*_*df* = 96_ = 1087.86; *p* < 0.001; *TLI* = 0.850; *CFI* = 0.924; *RMSEA* = 0.117, 95%-CI: 0.111–0.124). The residual matrix showed that the covariances at t_3_, t_4_ and t_5_ with measured values at t_i-2_ (i.e., t_1_, t_2_ and t_3_, respectively) were systematically underestimated for each of the four analyzed variables. Thus, predicting the subsequent values of the four variables required both their values at the preceding time point (t_i-1_, time lag = 1) and two time points prior (t_i-2_, time lag = 2). This proved to be necessary to model stability over time. After including the values at assessment time t_i-2_ as additional predictors for all four variables, the model fit proved to be good: *χ^2^*_*df* = 84_ = 247.67; *p* < 0.001; *TLI* = 0.972; *CFI* = 0.987; *RMSEA* = 0.051, 95%-CI: 0.044–0.059. Accordingly, the information in the variance–covariance matrix of all 20 analyzed variables can be described statistically appropriately if (i) each variable is regressed on the state of the respective variable itself at t_i-1_ and t_i-2_ (feature stability), and (ii) the state of the remaining variables at t_i-1_. Thus, this adaptation of the model structure ensures data compatibility of the model structure according to hypothesis 1. The multi-group CLPD model, in which the model parameters were determined separately for children and adolescents, showed a virtual identical model fit according to *TLI* = 0.971, *CFI* = 0.987, and *RMSEA* = 0.037 (95%-CI: [0.031–0.042]).

Based on the adapted model specification, the stability and reciprocal prediction between the analysis characteristics QoL, psychosomatic symptoms, externalizing and internalizing MH problems can therefore be validly analyzed. This applies both for the entire samples and after separating age groups. The latter is crucial for determining and contrasting moderation effects by means of multigroup analyses.

*Hypothesis 2 and 3*—*Moderation of cross-lagged predictions between* var*iables*: After restricting paths representing the time-shifted cross-prediction between the constructs to 0, the model fit significantly decreased (restriction in both groups simultaneously: Δχ^2^_*df* = 48_ = 281.13, *p* < 0.001; only children: Δχ^2^_*df* = 48_ = 212.61, *p* < 0.001; only adolescents: Δχ^2^_*df* = 48_ = 162.12, *p* < 0.001; Table 1). Accordingly, time-lagged cross-predictions must be included as essential components to adequately represent the data structure.

**Table 1:** Δχ^2^-values for nested model comparisons: models with restriction of the respective cross-predictions to 0 of the four time intervals for the according construct combinations vs. model with unrestricted parameter estimation.

Antecedent		Consequent					
	df	→ QoL	→ PSYSOM	→ EXT-MH	→ INT-MH	df	→ ALL
Total (*N* = 744)							
QoL →	3	**--**	9.32	2.66	11.66**	12	21.81*
PSYSOM →	3	30.41***	--	13.71**	26.01***	12	44.48***
EXT-MH →	3	9.40*	18.02**	--	6.09	12	23.95*
INT-MH →	3	25.74***	10.57*	2.19	--	12	32.60***
**ALL** →	**12**	**125.37** ^***^	**65.87** ^***^	**34.61** ^**^	**69.69** ^***^	48	281.13***
Children (*n* = 323)							
QoL →	3	--	4.25	8.89	3.54	12	**15.53**
PSYSOM →	3	20.41***	--	17.39***	25.87***	12	42.46***
EXT-MH →	3	5.35	32.29***	--	4.34	12	34.25**
INT-MH →	3	12.58**	9.58*	5.09	--	12	25.03*
**ALL** →	**12**	71.58*	62.70***	38.48***	53.41***	48	**212.61** ^***^
Adolescents (*n* = 421)—All variables parent reported							
QoL →	3	**--**	7.38	7.25	8.99	12	28.70**
PSYSOM →	3	10.18*	**--**	7.54	6.96	12	**17.80**
EXT-MH →	3	6.61	5.76	**--**	1.97	12	**14.44**
INT-MH →	3	14.51**	10.91*	3.60	**--**	12	24.96*
**ALL →**	**12**	58.00***	40.78***	**26.89**	25.87*	48	162.12***

Adolescents (*N* = 421)—QoL and HBSC-SCL self-reported; SDQ parent reported							
		**QOL** ^**SR**^	**HBSC** ^**SR**^	**SDQ-EXT**	**SDQ-INT**		
QoL^SR^→	3	--	14.22**	4.33	7.97	12	**30.55** ^**^
PSYSOM^SR^ →	3	15.14**	--	13.67**	16.29***	12	**25.44** ^*^
EXT-MH →	3	2.66	7.94	--	10.82*	12	**21.47** ^*^
INT-MH →	3	13.41**	13.69**	1.50	--	12	**20.79** ^*^
**ALL →**	12	74.23***	39.01***	**20.68**	47.42***	48	**186.67** ^***^

*p < 0.05; **p < 0.01; ***p < 0.001. QoL, Health related quality of life measured by the KIDSCREEN-10 Index; PSYSOM, Psychosomatic symptoms measured by the HBSC-SCL; EXT-MH/INT-MH, External/Internal mental health problems measured by the SDQ; SR, self-reported. Boldfaced: Overall tests.

To examine general moderating effects of the age-groups children vs. adolescents, each of the 48 cross-prediction paths were set equal between children and adolescents in an overall model (group-invariant estimation of the cross-predictions). Since these restrictions resulted in a significantly decreased model fit (Δχ^2^_*df* = 48_ = 167.84, *p* < 0.001), systematic differences for children and adolescents in the cross-predictions (i.e. moderating effects of age-groups; Hypothesis 3) have been confirmed. Accordingly, all results are calculated and presented separately for children and adolescents in the following.

In Table 1, row values provide information on the time-shifted predictions of the individual MH scales.

For children, QoL was found to be no time-shifted cross-predictor. If all cross-paths originating from QoL at t_1_, t_2_, t_3_ and t_4_ are restricted to 0 simultaneously, Δχ^2^_*df* = 12_ = 15.53 (*p* > 0.05) indicates no model deterioration. In contrast, psychosomatic symptoms (Δχ^2^_*df* = 12_ = 42.46, *p* < 0.001), externalizing (Δχ^2^_*df* = 12_ = 34.25, *p* < 0.001) and internalizing MH problems (Δχ^2^*_df = 12_* = 25.03, *p* < 0.001) proved to be cross predictors. Psychosomatic symptoms had significant predictive value for all other constructs (Table 1: → QoL: Δχ^2^_*df* = 3_ = 20.4, *p* < 0.001; → EXT-MH: Δχ^2^_*df* = 3_ = 17.39, *p* < 0.001; → INT-MH: Δχ^2^_*df* = 3_ = 25.87, *p* <. 001). Externalizing MH problems were predictive for psychosomatic symptoms (Δχ^2^_*df* = 3_ = 32.29, *p* < 0.001) and internalizing MH problems predicted QoL (Δχ^2^_*df* = 3_ = 12.58; *p* = 0.006) and psychosomatic symptoms (Δχ^2^_*df* = 3_ = 9.58; *p* = 0.022) at t_i + 1_.

**Figure 3:**
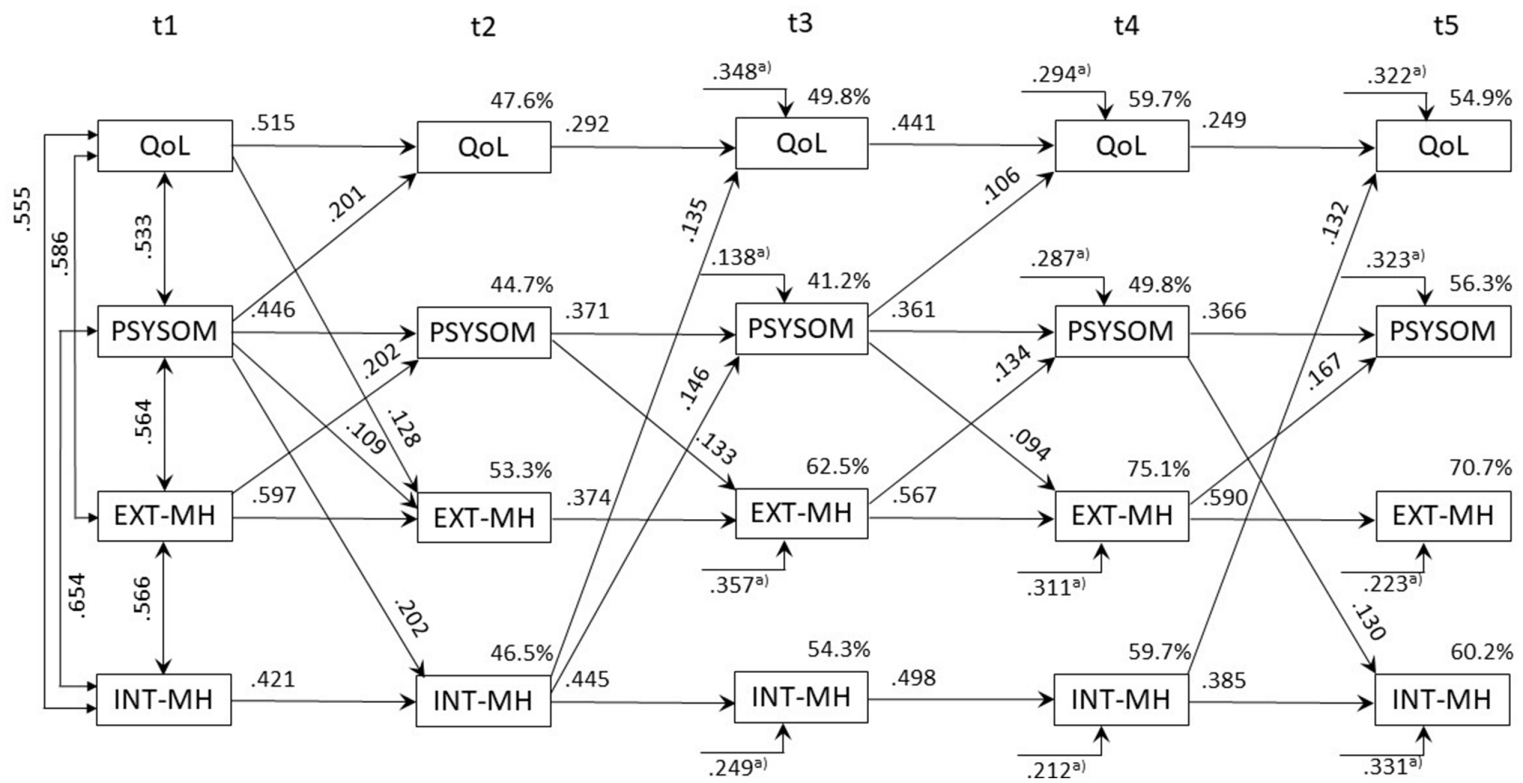
Correlations, standardized regression weights and variance explained in the cross-lagged-panel model for children (*n* = 323). Only significant values (*p* < 0.05) are displayed. QoL measured by the KIDSCREEN-10 Index; PSYSOM, Psychosomatic symptoms measured by the HBSC-SCL; EXT-MH/INT-MH, External/Internal MH problems measured by the SDQ. The endogenous error terms of the 4 variables are assumed to be correlated within each individual assessment time point t2, t3, t4 and t5. ^a)^ Regression weights for time-lag t_i-2_.

The column values in Table 1 provide information whether the respective column variable was systematically predicted by the other variable at t_i-1_. Here, it was examined for each variable whether the cross-regression paths directed towards the respective scale at t_2_, t_3_, t_4_ and t_5_ differed from 0. In children, QoL was predicted best (Δχ^2^_*df* = 12_ = 71.58, *p* < 0.001) with psychosomatic symptoms (Δχ^2^_*df* = 3_ = 20.41, *p* < 0.001) and internalizing MH problems (Δχ^2^_*df* = 3_ = 12.58, *p* = 0.006) being relevant antecedent scales (Hypothesis 2 confirmed for children). Psychosomatic symptoms as well as externalizing and internalizing MH problems proved to be mutually predictive: The systematic prediction of externalizing and internalizing MH problems were only due to psychosomatic symptoms (Δχ^2^_*df* = 3_ = 17.39 and 25.87, *p* < 0.001), whereas psychosomatic symptoms were predicted significantly by externalizing and internalizing MH problems (Δχ^2^_*df* = 3_ = 32.29, p < 0.001, and 9.58, *p* < 0.022). Figure 3 depicts the significant individual paths in the CLPD model.

In summary, for children QoL was not a cross-predictor over time, whereas psychosomatic symptoms, along externalizing and internalizing MH problems, showed significant cross-predictive relationships, with psychosomatic symptoms playing a central role.

**Figure 4:**
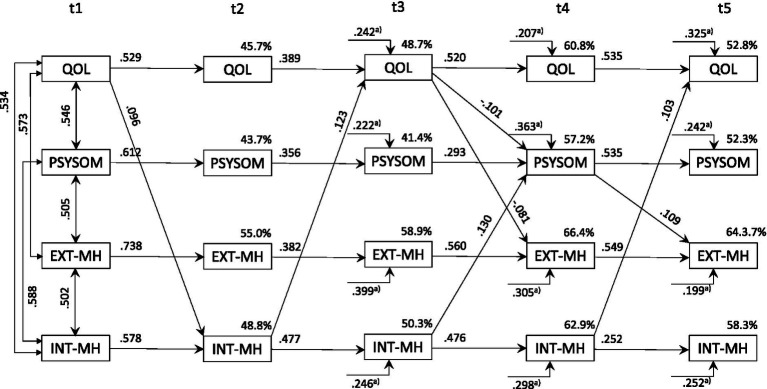
Correlations, standardized regression weights and variance explained in the cross-lagged-panel model for adolescents (*n* = 421; QoL^SR^ and HBSC^SR^ self-reported; SDQ: parent reported). Only significant values (*p* < 0.05) are displayed; QoL measured by the KIDSCREEN-10 Index; PSYSOM, Psychosomatic symptoms measured by the HBSC-SCL; EXT-MH/INT-MH, External/Internal MH problems measured by the SDQ. The endogenous error terms of the 4 variables are assumed to be correlated within each individual assessment time point t2, t3, t4, and t5. SR, Self-reported. ^a)^ Regression weights for time-lag t_i-2_.

Estimation of the CLPD model for adolescents based on parent-reported data revealed a considerably weaker cross-predictive structure (Table 1 and Supplementary Figure 1). In contrast to the sample of children, psychosomatic symptoms and externalizing MH symptoms did not prove to be significant cross-predictors for adolescents (Δχ^2^_*df* = 12_ = 17.80 and 14.44; *p* > 0.05). However, if self-reported QoL^SR^ and self-reported psychosomatic symptoms (PSYSOM^SR^) were used as model variables, self-reported psychosomatic symptoms also proved to be a significant cross-predictor for QoL^SR^ (Hypothesis 2 confirmed for adolescents), externalizing and internalizing MH problems (Δχ^2^_*df* = 3_ = 15.14, *p* = 0.002 / Δχ^2^_*df* = 3_ = 13.67, *p* = 0.003 / Δχ^2^_*df* = 3_ = 16.29, *p* < 0.001). In addition, QoL^SR^ predicted self-rated psychosomatic symptoms systematically (Δχ^2^_*df* = 3_ = 14.22, *p* = 0.003). The predictive effect of externalizing and internalizing psychological symptoms was evident for both the self-reported and the parent-reported data. Although the self- and parent ratings for both QoL (*r*_t1-t5_: 0.763, 0.794, 0.808, 0.842, 0.784) and psychosomatic symptoms (*r*_t1-t5_: 0.757, 0.821, 0.837, 0.851, 0.844) correlated very strongly at all five assessment points, CLPD predictions were considerably better overall when using self-rating data. Figure 4 shows the model structure for adolescents when self-reported psychosomatic symptoms and QoL^SR^ were used. In summary, for adolescents, the cross-predictive relationships were weaker in parent-reported data, but self-reported psychosomatic symptoms significantly predicted QoL, as well as externalizing and internalizing MH problems, with overall better model predictions based on self-reports.

For reasons of clarity, only the significant individual paths are shown in Figures 3, 4. A complete picture of all paths and their statistical significance is given in Table 2 (stability in the diagonal; cross-prediction in the off-diagonal) for all five assessment points. In addition, the significance of the moderation of age-groups for the individual paths can be obtained for all individual paths. Please note that the tests of the central cross-lagged prediction in this article were based on the data in Table 1, which enable a much more targeted test. Table 2 therefore only provides additional detailed information that is intended to make the overall data situation more transparent.

**Table 2:** Standardized regression weights from the prior (rows) to the subsequent (columns) measurement point in time (ti → ti+1) for children and adolescents.

	QoL t_i + 1_			PSYSOM t_i + 1_			EXT-MH t_i + 1_			INT-MH t_i + 1_		
	Children	Adolescents (P|S)	p(Δχ^2^)	Children	Adolescents (P|S)	p(Δχ^2^)	Children	Adolescents (P|S)	p(Δχ^2^)	Children	Adolescents (P|S)	p(Δχ^2^)
QoL												
t1	0.515***	0.505***| 0.529***	0.332	0.016	−0.038 | 0.067	0.468	0.128**	−0.011 | -0.011	0.025*	0.067	0.096* | 0.078	0.831
t2	0.292***	0.389*** | 0.478***	0.299	0.034	0.060 | 0.098*	0.697	−0.012	0.077 | 0.074	0.198	0.044	0.076 | 0.051	0.669
t3	0.441***	0.520*** | 0.420***	0.207	0.079	0.101* | 0.125**	0.516	0.050	−0.081* | -0.027	0.020*	0.053	0.057 | 0.076	0.931
t4	0.249***	0.535*** | 0.373***	0.801	−0.069	0.033 | 0.017	0.155	0.039	−0.002 | -0.017	0.472	−0.020	−0.029 | 0.000	0.909
PSYSOM												
t1	0.201***	0.089 | 0.118**	0.167	0.446***	0.612*** | 491***	0.001**	0.109*	0.009 | 0.068	0.136	0.202***	0.044 | 0.086*	0.034
t2	0.034	0.058| 0.083	0.914	0.371***	0.356***| 0.422***	0.869	0.133*	−0.015 | -0.012	0.017*	0.092	0.029 | 0.122**	0.337
t3	0.106*	0.060 | 0.070	0.395	0.361***	0.293***| 0.379***	0.694	0.094*	0.039 | 0.002	0.293	0.088	0.061 | 0.069	0.590
t4	0.090	0.093 | 0.060	0.801	0.366***	0.535***|0.509***	0.160	0.032	0.109* | 0.123**	0.387	0.130***	0.087 | 0.059	0.218
EXT-MH												
t1	0.069	0.079 | 0.059	0.829	0.220***	0.083 |0.062	0.125	0.597***	0.738*** | 0.759***	0.132	0.098	0.048 | 0.130**	0.496
t2	0.048	0.015 | -0.012	0.672	−0.055	0.070 | 0.074	0.702	0.374***	0.382*** | 0.436***	0.927	0.015	−0.041 | -0.035	0.417
t3	0.012	0.035 | 0.030	0.665	0.134*	0.027 | 0.020	0.252	0.567***	0.560*** | 0.566***	0.743	0.020	0.003 | 0.008	0.812
t4	0.089	0.058 | 0*.006*	0.724	0.167***	−0.004 | 0.062	0.015*	0.590***	0.549*** | 0.564***	0.526	0.047	−0.007 | 0.018	0.378
INT-MH												
t1	−0.002	0.057 | 0.006	0.405	0.083	0.063 | 0.135**	0.958	−0.031	0.010 |-0.029	0.552	0.421***	0.578*** | 0.536***	0.006^**^
t2	0.135*	0.123^*^ | 0.062	0.870	0.146*	0.054 | -0.029	0.335	0.045	0.003 | -0.008	0.495	0.445***	0.477*** | 0.466***	0.688
t3	0.031	0.071 | 0.119^**^	0.095	−0.031	0.130***|0.103*	0.010*	0.078	0.064 | 0.035	0.013*	0.498***	0.476*** | 0.490***	0.882
t4	0.132*	0.103^*^ | 0.055	0.675	0.077	−0.025 | 0.018	0.175	0.021	0.045 | 0.000	0.751	0.385***	0.252*** | 0.460***	0.223

*p < 0.05; **p < 0.01; ***p < 0.001. Boldfaced: significant values. Δχ^2^: χ^2^-difference of nested models; QoL, Health Quality of life (KIDSCREEN-10 Index); QoL, Health related quality of life measured by the KIDSCREEN-10 Index; PSYSOM, Psychosomatic symptoms measured by the HBSC-SCL; EXT-MH/INT-MH, External/Internal mental health problems measured by the SDQ.; P, Parent-reported; S, Self-reported; Values in the diagonal: Stability of characteristics from ti to ti + 1. Off-diagonal: Cross prediction between characteristics from t_i_ to t_i + 1_.

Table 3 shows the results of the invariance test of parent-reported data based on CFA modeling. According to the Δχ^2^-test, all four constructs analyzed exhibit a significantly poorer model fit under the assumption of time-invariant loading structures. In contrast, the parsimony-adjusted measures *PCFI* identifies the measurement-invariant loading structure as consistently better for all four constructs (invariant: 0.782, 0.778, 0.807, 0.794 vs. free: 0.758, 0.757, 0.785, 0.751). The *BIC* supports this for the measurement of QoL by the KIDSCREEN as well as the external and internal MH of the SDQ (invariant: 4776.16, 4132.87, 1398.46 < free: 4944.22, 4247.78, 1449.87). However, there was a lower value for psychosomatic symptoms according to the HBSC-SCL for free loading estimates (3022.57 < invariant: 3046.67). According to the *ΔNFI*, the loss of data fit due to the invariance assumption is also markedly higher for this scale at 0.015 than for the others (0.003–0.006).

**Table 3:** Nested model comparisons to test measurement invariance (tau-equivalent measurements, parent-reported data) for each of the four analyzed constructs.

	χ^2^	df	*p*	RMSEA	CFI	PCFI	BIC	ΔNFI	Δχ^2^	df	*p*
Qol											
Free	3,615,20	1,074	<0.001	0.056	0.865	0.758	4944.22	0.003	67.98	36	0.001
Invariant	3,683,17	1,110	<0.001	0.056	0.863	**0.782**	**4774.16**				
PSYSOM											
Free	1905.14	651	<0.001	0.051	0.907	0.757	**3022.57**	0.015	209.23	28	<0.001
Invariant	2114.37	679	<0.001	0.053	0.893	**0.778**	3046.67				
EXT-MH											
Free	2685.87	1,066	<0.001	0.048	0.902	0.785	4247.78	0.006	123.12	36	<0.001
Invariant	2,988,99	1,102	<0.001	0.048	0.897	**0.807**	**4132.87**				
INT-MH											
Free	638.22	335	<0.001	0.035	0.972	0.751	1449.87	0.003	32.92	20	0.034
Invariant	671.14	355	<0.001	0.035	0.971	**0.794**	**1398.46**				

Δχ2, χ2-difference of nested models; QoL, Quality of life measured by the KIDSCREEN-10 Index; PSYSOM, Psychosomatic symptoms measured by the HBSC-SCL; EXT-MH/INT-MH, External/Internal mental health problems measured by the SDQ. Boldfaced: superior values in comparison of the two models.

## 4 Discussion

The aim of the present study was to investigate reciprocal time-lagged cross predictions between psychosomatic symptoms, internalizing and externalizing MH problems as well as QoL in children and adolescents from the initial phase of the COVID-19 pandemic in spring 2020 to the phase with significantly relaxed protective measures in autumn 2022. The stability over the five assessment points and cross-lagged predictions of MH indicators and QoL could be adequately modeled by the CLPD (hypothesis 1 confirmed), with a significantly different prediction structure for children and adolescents (hypothesis 3 confirmed). Consistent with hypothesis 2, the results indicate a time-lagged cross prediction of MH constructs on QoL in children: QoL was most strongly cross-predicted by psychosomatic symptoms (t_1_ → t_2_, t_3_ → t_4_) and internal MH (t_2_ → t_3_, t_4_ → t_5_), but QoL itself had no significant cross-predictive value for psychosomatic symptoms as well as internal and external MH.

These results for children are consistent with findings that psychosomatic symptoms explain between 27 and 50% of the variance in children’s QoL (Svedberg et al., 2023). In particular, depressive symptoms, concentration and sleep problems as well as stomach aches proved to be associated with QoL. An overlapping pattern of anxiety, depression, and psychosomatic symptoms associated with low QoL outcomes was reported for children and adolescents (Barbieri et al., 2023). In both studies, psychosomatic symptoms and mental problems are discussed as causes for the development of QoL despite the cross-sectional study design (see also: Celebre et al., 2021; Kauhanen et al., 2023; Tal-Saban and Zaguri-Vittenberg, 2022). Such causal assumptions are also proposed for longitudinal study findings (Kauhanen et al., 2023; Orban et al., 2024; Otto et al., 2017; Wolf and Schmitz, 2023; Montero-Marin et al., 2023). Through the integrative analysis of the cross-sectional and longitudinal data from the COPSY study, an improved evidence base for the presumed time-lagged direction with the help of CLPD modeling has been achieved (Selig and Little, 2012; Singer and Willett, 2023; Finkel, 1995).

For adolescents, the result structure is less clear. While for parent-reported psychosomatic symptoms no time-shifted cross-prediction could be revealed, the self-reported psychosomatic symptoms are significant cross-predictors for all other constructs. In research on MH (Barbieri et al., 2023; Celebre et al., 2021; Larsen et al., 2022; Mesman et al., 2021; Svedberg et al., 2023; van der Laan et al., 2021) and its consequences (Achenbach et al., 1987; de Los et al., 2015) the validity of parent-reported indicators of adolescents’ health is critically discussed. Due to the development of individuation and autonomy, adolescents become more detached from their parents and relations with peers become more important. Hence, parents may no longer be the most appropriate informants regarding MH and QoL of adolescents (Achenbach et al., 1987; de Los et al., 2015; Piehler et al., 2020; Waters et al., 2003). In general, children’s self-reports show a better agreement with objective indicators, especially for internalizing symptoms such as anxiety or depression, which can only be partially observed based on behavioral indicators (de Los et al., 2016). Meta-analyses show a low to moderate correlation between self- and parent ratings, with parents tending to assess externalizing problems (e.g., aggressive behavior) more reliably, while children and adolescents report internalizing symptoms better (Achenbach et al., 1987; Rescorla et al., 2013). In addition, developmental factors influence validity: younger children tend to be less reliable in their self-assessments than older adolescents, who have higher metacognitive abilities and more stable self-perception (Van Roy et al., 2010). Contextual factors play a role as parent ratings usually reflect behavior at home, while self-reports tend to reflect experiences in different social contexts).

In this study, effects across measurement points were tested by simultaneously restricting the cross-paths of t_1_ → t_2_, t_2_ → t_3_, t_3_ → t_4_ and t_4_ → t_5_ for each variable combination (e. g. PSYSOM → QoL) to zero. The cross-predictions were thus tested independently of the respective time interval (Table 1). This was considered appropriate because no *a priori* hypotheses could be derived why the time-lagged effects between MH constructs and QoL should differ among the different time intervals (e.g., due to interval specific COVID-19 infection incidence or protective measures). Accordingly, the simpler hypothesis of the time-invariant dependency structure was considered appropriate (Lüdtke and Robitzsch, 2022; Kline, 2023). Note, however, that if an interval-invariant overall effect proved to be significant (Table 1), this did not necessarily imply that all four individual paths were significant in the individual test (Table 2). At first glance, this seems to lead to contradictory results, especially for adolescents: e.g., a time-shifted cross-prediction of self-reported psychosomatic symptoms on QoL^SR^ could be confirmed across assessment points (Table 1). However, only for the first individual paths t_1_ → t_2_ from self-reported psychosomatic symptoms on QoL^SR^ proved to be significant (Table 2, Figure 4). The path-specific findings in Table 2 on time-shifted cross-predictions possess correspondingly less test power and are thus much more error-prone (enhanced β-error probability) due to reduced test power (Ellis, 2010) than the cross-measurement-time comparisons in Table 1. Only the latter reveal the general high impact of the cross-predictions in the CLPD model.

The analyses were conducted using the manifest scale values of the measurement instruments. Although the same aggregation rule for the scale values is used for each measurement time point, violations of the measurement invariance may influence the model estimates (in particular trait stability and cross-predictions) due to measurement time point-dependent changes in the construct-item associations (Putnick and Bornstein, 2016; Speyer et al., 2023). The analyses of measurement invariance of the individual measurement scales across the five measurement time points proved to be at most weak. Thus, a stable association of the measurement indicators with the characteristics to be measured has been confirmed. Only psychosomatic symptoms according to the HBSC-SCL show clearer effects that suggest a measurement invariance violation. Accordingly, it must be assumed that the same measured characteristic values may be based on different patterns of psychosomatic symptoms for different measurement times. Since corresponding effects, especially when using the same measurement procedure for different measurement times, do not fundamentally call validity into question (Robitzsch and Lüdtke, 2023), reference is made to this only in the sense of a future research desideratum. It would be interesting to shed more light on how symptom patterns and their changes, which shape the health situation of children and adolescents, depend on specific life circumstances that affect their everyday behavior.

### 4.1 Limitations of the study

Although the fundamental strength of CLPDs is to determine causal effects despite the lack of randomized control, assumptions must be considered in the causal interpretation of the time-delayed cross-predictions (Finkel, 1995; Robitzsch and Lüdtke, 2024; Selig and Little, 2012; Singer and Willett, 2023). Significant directional cross-predictions only conclusively reflect a causal direction if no confounding characteristics are missing in the model. Consequently, the fact that the analyses were modeled independently of other health-related aspects must be considered a limitation (Kenny, 2004). Accordingly, the diagnostic-prognostic interpretation of the model findings, which is not affected by this problem of “omitted variables” (Lucas, 2023; Selig and Little, 2012), was prioritized. For example, in a diagnostic sense, it has been shown for children that MH aspects predict QoL with a time delay and not vice versa during the pandemic. However, this should not be regarded as conclusive confirmation of MH symptoms as a definitive cause of QoL development.

The model variables were analyzed as manifest scale scores of established instruments. Generally, analyzing model variables as latent constructs would provide measurement error-adjusted and thus more unbiased estimates of model parameters (Bollen, 2002). However, latent modeling was not possible due to the considerably higher model complexity. The CLPD with manifest scale scores is already quite complex with 210 parameters to be estimated. The use of latent modeling would have increased the complexity problem unacceptably (e.g., negative degrees of freedom when using the individual items) (Kline, 2023). Ceiling effects prevailed for the SDQ questionnaire, which limited the variance of the assessed characteristics and resulted in asymmetric distributions. However, skewness was below 3 and kurtosis was below 8 for all variables, indicating appropriate distributions for valid parameter estimates and significance testing (Kline, 2023).

Nested age-group comparative tests were only based on parental proxy indicators. Since the supplementary analysis for adolescents with self-reported QoL and psychosomatic symptoms revealed considerable discrepancies in the self-reported vs. parent-reported data, it would have been beneficial to also assess data on self-reported internalizing and externalizing MH problems (de Los et al., 2015; Piehler et al., 2020; Waters et al., 2003). Since QoL is defined from the subjective perspective of experience, additional self-reported QoL indicators would also have been useful for children (Aaronson et al., 2002). Finally, although the proportion of complete data sets of 46.9% in the approximately 2.5-year course of the survey is high and only minor indicators of systematic drop out were found, it cannot be ruled out that systematic dropout of families and adolescents may have affected the results (violation of the MCAR-assumption in CCA; Enders, 2008; Graham, 2009; Wirtz, 2004). It would have been beneficial to have recorded informative covariates of the drop-out at the individual measurement points in order to be able to fulfill the MAR assumption. This would have permitted the inclusion of all families in the analysis despite the missing data by applying the probability-based FIML algorithm. This approach would have allowed possible biases to be taken into account and corrected. Accordingly, no missing data imputation was carried out. Aspects of systematic drop-out were addressed in connection with the extended sample description. This aspect concerns the external validity and generalizability of the findings. Despite the population-based approach, potential distortions associated with higher age and higher social status may impair the generalizability of the study results. In future research, systematic methods, such as Dillman’s Tailored Design method (Dillman et al., 2024) or the INTACT-RS model (Lander et al., 2023), should be used, integrating well-founded approaches to comprehensively ensure the most representative sample possible and to avoid drop-out.

Furthermore, a more comprehensive representation of heterogeneous socioeconomic and sociocultural characteristics would be important to analyze and differentiate the moderating effects on the analyzed CLPD structure. E.g., lower-income families face greater challenges in accessing resources, while unstable home environments may increase stress and insecurity (Lazzarino et al., 2014). Disruptions to education have widened academic gaps and strained peer relationships that are important for social and emotional development (Farrell et al., 2023; Gniewosz, 2023). Community support systems and cultural attitudes toward MH may further affect recovery and resilience (Vandrevala et al., 2024). In future studies it would be interesting to determine protective aspects of coping and resilience from a salutogenetic perspective to provide children and adolescents with targeted support, both individually and through social frameworks (esp. in educational institutions) (Groß et al., 2024). The COPSY study examined the development of MH and QoL under the specific conditions of the COVID-19 pandemic. However, uncertainties remain regarding the generalizability of the findings. A key issue to consider is whether these results apply only to times of significant restrictions on daily and social life or if they can also be extended to non-pandemic contexts. Future research should pay special attention to the influence of structural conditions and life circumstances on MH and QoL to better delineate the applicability of these findings.

Finally, it must be taken into account that during the pandemic measures to prevent infection as well as social conditions significantly influenced the lives of children and adolescents over time. Schools may have closed abruptly, and the necessity of physical/social distancing varied considerably due to the risk of infection and associated political measures. In addition to the temporal variability, regional differences must also be considered, since living conditions related to infection control were not uniform throughout Germany. In order to be able to interpret the parameter estimates in the CLPD appropriately and to provide a framework for them, it would be very informative to characterize appropriate contextualizations (Kuiper and Ryan, 2018; Sukpan and Kuiper, 2023). It would be very valuable if more targeted hypotheses about the relationship between contextual circumstances that change over time and the dependencies between the variables could be tested. However, a correspondingly differentiated and meaningful documentation of the contextual conditions could not be guaranteed, and this would have been difficult to achieve due to the specific conditions during the pandemic. Therefore, comparing the strength of the relationships between the survey dates has been avoided when interpreting results. Note that cross-relations between the variables were treated as interval independent (i.e., the cross-lagged predictions are tested by equating the paths in all four intervals t_1_ → t_2_, t_2_ → t_3_, t_3_ → t_4_; t_4_ → t_5_ simultaneously in the overall model). Inferences regarding the significance of interval-specific contextual factors are not permissible in this form and would be speculative. Although the intervals between the measurement points should be kept as even as possible, the intervals varied between 6 and 8 months. This variation may have influenced the estimate of the strength of trait stability and cross-prediction (Orth et al., 2021; Selig and Little, 2012).

## 5 Conclusion

In summary, the longitudinal data of the German COPSY study allowed to systematically analyze the time-delayed predictions of MH indicators and QoL in children and adolescents during the course of the COVID-19 pandemic. Evidence from cross-sectional as well as longitudinal studies on the possible impact of psychosomatic symptoms and mental problems for the future development of QoL could be improved considerably by the more internally valid CLPD modeling. Future research should particularly focus on characteristics potentially confounded with the analysis characteristics and on interventional elements in order to further increase the conclusiveness and internal validity of the evidence for directional effect relationships. While our findings suggest that MH can be considered an antecedent predictor of the development of QoL in children, in adolescents, QoL itself can also be regarded as a determinant or leading indicator of MH development. As it is challenging to examine the natural development of MH and QoL in children and adolescents - i.e. critical prerequisites and subsequent consequences - the study findings offer very promising insights to advance the understanding and support processes underlying MH development in children and adolescents.

## Data Availability

The raw data supporting the conclusions of this article will be made available by the authors, without undue reservation.

## Glossary

AMOS: analysis of moment structure
CCA: complete-case-analysis
CFI: comparative fit index
CLPD: cross-lagged-panel design
COPSY: COVID-19 and psychological health study
COVID-19: corona virus disease 2019
EXT-MH: external mental health problems
FIML: full-information-maximum-likelihood
HBSC-SCL: health behavior in school-aged children symptom checklist
INT-MH: internal mental health problems
MAR: missing-at-random
MCAR: missing-completely-at-random
MH: mental health
ML: maximum likelihood
PSYSOM: PSYSOM psychosomatic symptoms
QoL: quality of life
RMSEA: root mean square error of approximation
SEM: structural equation model
SDQ: strength and difficulties questionnaire
SR: self-reported
TLI: Tucker-Lewis Index
