# Supplementary figures and images for: Reciprocal impact of mental health and quality of life in children and adolescents—a cross-lagged panel analysis
Source: Front Psychol. 2025 Mar 26;16:1444524. doi: 10.3389/fpsyg.2025.1444524 (PMC11983161; doi:10.3389/fpsyg.2025.1444524)

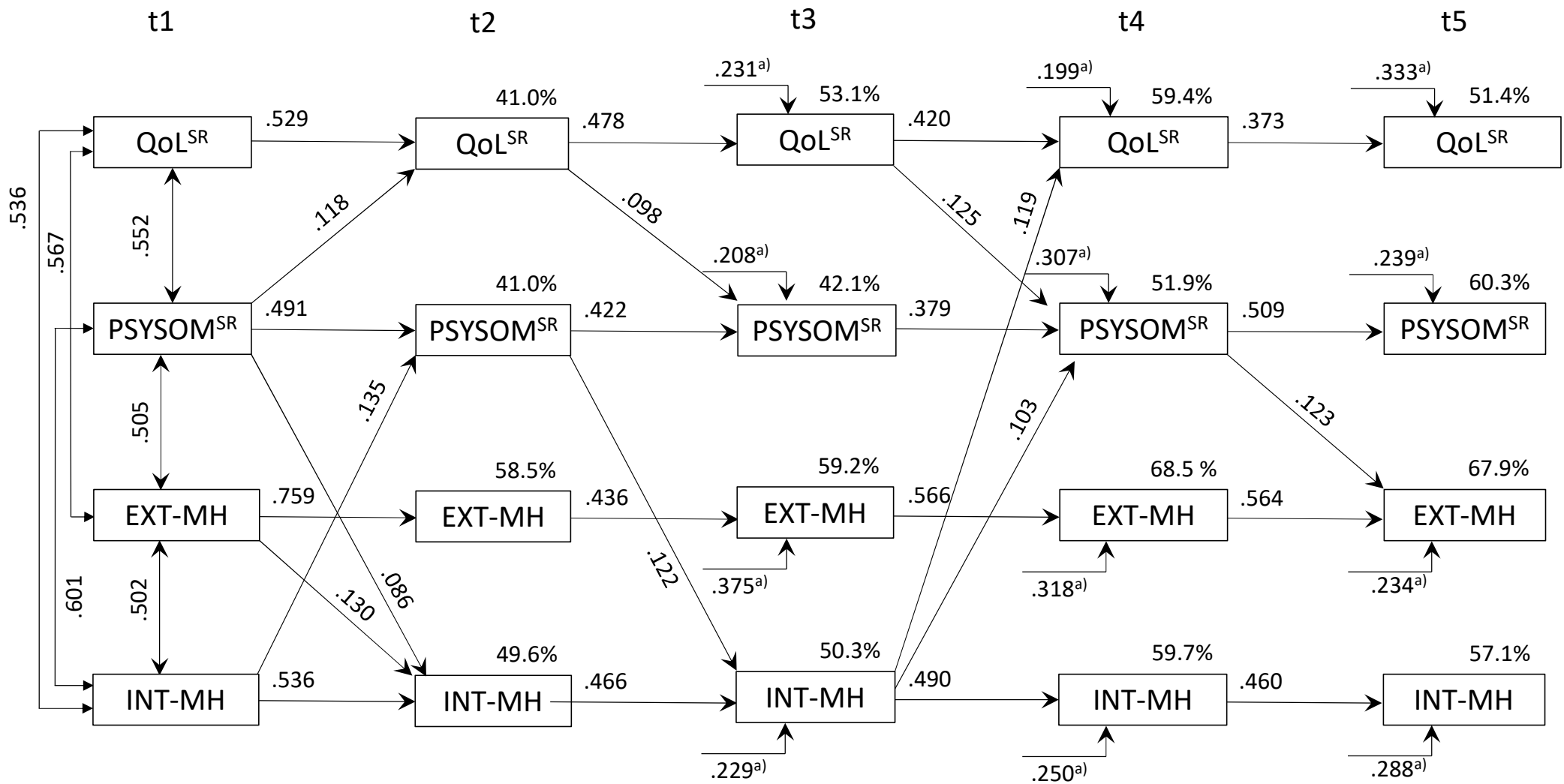

Supplement: Supplementary file 1 [file Image_1.pdf]
